# Role of Ultrasonography Supplemented by Sialendoscopy in Submandibular Steinstrasse Sialolithiasis

**DOI:** 10.7759/cureus.20286

**Published:** 2021-12-08

**Authors:** Ravikanth Reddy

**Affiliations:** 1 Radiodiagnosis, St. John's Hospital, Bengaluru, IND

**Keywords:** recurrent sialadenitis, steinstrasse, submandibular sialolithiasis, sialoendoscopy, ultrasonography

## Abstract

The submandibular gland is the most common major salivary gland vulnerable to sialadenitis secondary to sialolithiasis. We report a case of submandibular steinstrasse causing sialadenitis in a 45-year-old male and describe the appearances on high-resolution ultrasonography. Endoscopic-assisted excision of calculi was done. Post-operative recovery was uneventful and the patient was discharged after one week. The patient has been on follow-up for six months with no complaints of recurrence. Multiple stacked calculi within the Wharton’s duct is an exceedingly rare occurrence. Steinstrasse creates a dilemma of choice for the intended surgical approach during calculi extraction from the Wharton’s duct. Endoscopic guided calculi extraction may be ideal for distally placed calculi along the course of the duct. Submandibular steinstrasse can be a possibility when electrohydraulic or pneumatic techniques have been deployed.

## Introduction

Sialolithiasis is the deposition of calcific concretions composed of calcium and phosphate salts within major/minor salivary glands [1]. Wharton’s duct coursing through the submandibular gland is the most vulnerable site for calculi formation due to the alkaline pH of the mucus and tortuosity of the duct [2]. The submandibular duct arises from the deep part of the gland from the floor of the mouth along the lateral side of the frenulum linguae and this location allows for retrograde infection of the gland by oral flora leading to recurrent sialadenitis. Submandibular steinstrasse refers to a column of stone fragments causing complete blockage of the duct and this report depicts its sonological appearance. This steinstrasse occurrence in Wharton’s duct of the submandibular gland may cause a predicament in the choice of surgical approach while performing sialoendoscopic surgery. Intraglandular sialolith formation is rare and may require submandibular sialadenectomy [3]. However, correlating clinical and radiographic findings is important in the determination of the precise location to indicate the right treatment for the individual patient. 

## Case presentation

**Figure 1:**
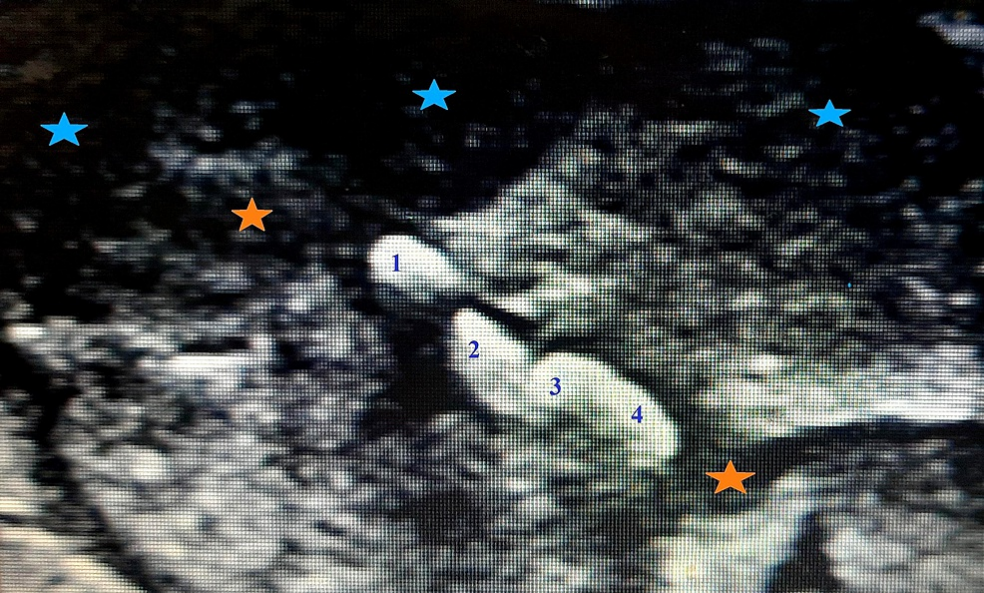
High-resolution ultrasonography image of the submandibular gland demonstrating steinstrasse in the Wharton’s duct causing mild dilatation consistent with features of submandibular sialolithiasis. Note the stacked calculi (numbers), dilated Wharton’s duct with sludge (orange stars), and hypoechoic areas (blue stars) within the submandibular gland consistent with features of sialadenitis.

A 45-year-old gentleman presented to the surgery department with complaints of recurrent pain and swelling in the right submandibular region for two months. On clinical examination, the skin was erythematous with tenderness in the right submandibular gland on palpation and the floor of the mouth was elevated on the right. High-resolution ultrasonography of the neck and submandibular region was requested. On ultrasonography, the right submandibular gland was atrophied and showed no increase in vascularity on color Doppler consistent with features of chronic sialadenitis (Figure 1); approximately 8-10 calculi in number measuring 6-8 mm in size have formed steinstrasse in the Wharton’s duct causing its mild dilatation consistent with features of sialolithiasis. On high-resolution ultrasonography, a diagnosis of sialolithiasis with sialadenitis of the submandibular gland was made. The patient was referred to the department of maxillofacial surgery for further management. Endoscopic-assisted excision of calculi was done. Post-operative recovery was uneventful and the patient was discharged after one week. The patient has been on follow-up for six months with no complaints of recurrence. The patient has given written informed consent to publish his case and clinical images.

## Discussion

The submandibular gland is most commonly affected (80-90% of cases) in sialolithiasis followed by the parotid gland. Factors such as long and tortuous course of the Wharton’s duct, salivary flow against gravity and higher mucin content in saliva produced by the submandibular gland are responsible for the high incidence of submandibular sialolithiasis [4]. Salivary stasis secondary to sialolithiasis causes sialadenitis. Incomplete obstruction of the Wharton’s duct leading to recurrent pain aggravated by chewing, complete obstruction causing constant pain, dilatation of the Wharton’s duct proximal to the site of obstruction and edematous submandibular gland or steinstrasse causing fibrosis and atrophy of the gland secondary to long standing obstruction are features of submandibular sialolithiasis [5].

Radiological investigations advocated in patients with salivary gland sialolithiasis include sialography, occlusal radiographs, orthopantomogram, ultrasonography, CT, and MRI [6]. However, ultrasonography can accurately confirm the length, total dimension and number of calculi in the submandibular duct thus aiding in decision making regarding the route and choice of surgical approach [7]. Sialography is a radiography technique wherein a radio-opaque dye is injected into the salivary duct to visualize the duct system and for sialolith detection. However, sialography is contraindicated in the event of acute infection due to the possibility of idiosyncratic reaction secondary to contrast medium injection. Furthermore, sialography is not indicated when there is strong clinical suspicion of a sialolith located in the distal portion of the duct, as the salivary duct calculus may be mobilized more proximally by the contrast medium, which might further complicate its removal [8]. However, high-resolution ultrasonography remains the diagnostic modality of choice in sialolithiasis.

Calcified intraglandular lymph node, glandular phlebolith, calcified hemangioma and palatine tonsilloliths are included in the differential diagnoses of sialolithiasis. Complications related to submandibular sialolithiasis are abscess formation, Ludwig angina, and intraoral fistula to the neck [9].

Extracorporeal shockwave lithotripsy and endoscopic intracorporeal shockwave lithotripsy have replaced the conventional submandibular gland surgery for sialolithiasis. Sialendoscopy can be used as a diagnostic and interventional modality for the retrieval of sialoliths from ducts of major salivary glands [10]. Transoral sialolithotomy with sialadenectomy or sialodochoplasty is the treatment method of choice for larger salivary gland calculi, while sialoendoscopy and extracorporeal short-wave lithotripsy have emerged as effective alternatives to conventional surgical excision for the treatment of smaller calculi. Procedures for fragmentation of sialoliths involving pneumatic and electrohydraulic devices have to be executed with caution due to the high risk of ductal injury. Smaller intraductal calculi extraction with the added benefit of biopsy may be obtained with sialendoscopy, which has the advantage of being both a diagnostic and an interventional procedure [11]. However, there is a high chance of fracture of larger calculi in the gland followed by likely dissemination of fragments into the gland parenchyma during the sialoendoscopy procedure, which is a matter of grave concern. 

## Conclusions

This case report describes a rare ultrasonography appearance of submandibular steinstrasse and stresses the fact that this rare clinical phenomenon should be included in the differential diagnosis in patients with recurrent sialadenitis. The report deserves a special mention as it describes an unusual occurrence of multiple stacked calculi within the Wharton’s duct creating a dilemma for the choice of surgical approach in submandibular sialolithiasis. In conclusion, ultrasonography can be used as a gold standard investigation of choice for pre-procedural assessment of patients with submandibular steinstrasse. Submandibular steinstrasse can also be a possibility when electrohydraulic or pneumatic techniques have been deployed for large sialolith fragmentation in cases with intractable pain. Therapeutic intervention is virtually made possible in all cases of sialolithiasis when ultrasonography is supplemented by sialendoscopy.
